# Human Health Risk Assessment of Cd, Cu, Pb and Zn through Consumption of Raw and Pasteurized Cow’s Milk

**Published:** 2018-08

**Authors:** Soheil SOBHANARDAKANI

**Affiliations:** Dept. of the Environment, College of Basic Sciences, Hamadan Branch, Islamic Azad University, Hamadan, Iran

**Keywords:** Food safety, Metal contamination, Health risk index, Milk

## Abstract

**Background:**

The analysis of the residual contents of heavy and toxic metals in foodstuff especially milk could be an important indicator of safety, quality, and level of pollution of the region in which the milk was produced. Therefore, the present investigation was carried out to assess human health risk of residues levels of four metals (Cd, Cu, Pb, and Zn) through consumption of raw and pasteurized cow’s milk.

**Methods:**

In this analytical-observational study, totally 72 samples of raw and pasteurized cow’s milk samples were collected from market basket of Hamadan City, western Iran in 2014. After preparation and processing the samples in the laboratory, the concentration of metals were determined using inductively coupled plasma (ICP-OES). Moreover, all statistical analyses were performed using the SPSS statistical package according to Shapiro-Wilk test for normality, One Way ANOVA (Duncan Multiple Range Test), Independent t-test and Pearson’s correlations.

**Results:**

The mean concentrations (μg/kg) of Cd, Cu, Pb and Zn in raw milk samples were 0.36±0.28, 9.77±3.91, 32.83±20.80 and 253.70±87.96, respectively and in the pasteurized milk samples were 5.57±9.33, 8.41±5.99, 25.54±26.50 and 90.12±91.52, respectively. HRI values in adults and children via consumption of raw and pasteurized cow’s milk were within the safe limits (HRI < 1).

**Conclusion:**

Considering the serious contamination of some samples of raw and pasteurized milk by Cd, Pb and Zn, a control of heavy metals content during the whole production processing of milk must be applied.

## Introduction

The rapid development of our society in the past few decades, especially with priority of industrial activities, mining, fuel combustion, development of mechanized cultivation and consequently the use of large amount of agricultural inputs without attention to the their environmental and health impact, caused a wide distribution of heavy metals and other hazardous pollutants in the environment and finally in the trophic chain (1, 2).

The products with animal origin specifically milk and dairy products play an important role in human diet and acquire a special significance in infant nutrition considering their protein and mineral contents essential (1). Therefore, entry of high levels of heavy metals into the dairy products could be a potential risk, serious diseases and public health problems (3–5) and for this reason the determination of the heavy metals contents in livestock’s milk could be an important indicator of the hygienic condition of this product and also the level of pollution of the area in which the milk was produced (3, 6, 7).

Cadmium is toxic and non-essential for human health and accumulates principally in the kidneys and liver. Metal industries and sewers are known as the most important sources of Cd pollution (8).

Copper is one of most abundant trace elements with vitamin-like impact in human body and living systems and found in a wide range of foods we eat such as nuts, fruits, vegetables, red meat and shellfish (9, 10).

Lead can enter the food chain through contaminated soil. Lead poisoning can affect the blood circulation, kidneys, and peripheral and central nervous systems especially in adults (11). Furthermore, infants and children are at a particularly high risk for neurotoxic and developmental disorders due to this toxic element (12, 13).

However, zinc plays an important role in cancer etiology and harms some physiological activities, but this element known as a functional and structural element for human health (14, 15).

The human health risk assessment requires identification, collection, and integration of information on the chemicals health hazards, exposure of human to the chemical and relationships between exposure, dose and adverse effects (16). In this regard, a human potential health risk assessment is the process to estimate the nature and possibility of adverse health effects in humans exposed to toxins and chemicals in polluted environmental media, now or in the future (17).

The survey of potential health risks is done by compared the acceptable daily intake (ADI) of trace elements with the provisional tolerable weekly intake (PTWI) or oral reference dose (RfD). According to the standard methods (USEPA), the risk of chronic-toxic effects (health risk index (HRI) or target hazard quotients (THQ)) is described as the ratio of the dose resulting from exposure to site media to the dose believed to be safe, even in sensitive individuals i.e. children and elderly. Here, the HRI < 1 shows that no significant risk of chronic-toxic effects exists, while, the HRI > 1 shows that chronic-toxic effects may occur (18, 19).

In view of the concern for food safety, this study was conducted to analyze the Cd, Cu, Pb, and Zn contents in raw and pasteurized cows’ milk marketed Hamadan City, western Iran, in 2014.

## Materials and Methods

### Reagents

Standard stock solutions of analyzed metal ions at the concentration of 1000 ppm were used to prepare working solutions after appropriate dilution. Standard solutions were of analytical grade (Merck, Darmstadt, Germany). Distilled deionized water was used in all dilution procedures

### Sampling and Sample Analysis

In this study, a total of 36 samples of raw (from the 12 dairies) and 36 samples of pasteurized (from 12 brands) cow’s milk were collected from market basket of Hamadan City, western Iran in 2014. Milk samples were collected in sterile polypropylene bottles of 50 ml and they were frozen under −20 °C until their analysis (1).

In the laboratory, to obtain the total dehydration of samples, all milk samples were lyophilized. Moreover, for the acid digestion of samples, a microwave system was used. Then 10.0 ml of HNO_3_ (65%) was added to a dry sample of 0.5 gr of the lyophilized material. The solution was kept in the microwave for two periods of 5 min and 10 min to 100 °C and 175°C, respectively with an intermediate depressurization. The solution was kept to 4 °C up to the moment of its valuation (1, 3). The analysis of Cd, Cu, Pb, and Zn were performed by ICP-OES (Varian, 710-ES) in μg/kg. To control the accuracy of the experiment, a multi-element standard solution (Merck) with different concentrations of Cd, Cu, Pb and Zn (0.2, 1, 10, 50, 100 and 500 μg/L) were used for the calibration. For the half of the measurement, three controls of each metal are used, a blank and a standard of 10 μg/L (similar to the calibration) (1).

### Human Health Risk Assessment

For human health risk assessment the average daily intake of metal (DIM) was calculated using the equation 1 (20–22):

(1) $$\mathrm{DIM}=\frac{C\;\text{metal}\times C\;\text{factor}\times D\;\text{food}\;\text{intake}}{B\;\text{average}\;\text{weight}}$$,

where C_metal_, C_factor_, D_food intake_, and B_average weight_ are the heavy metal contents in milk (μg/kg), conversion factor (0.085) (21), average daily intake of milk (0.14 kg per person per day) (23), and average body weight (15.0 kg for children and 70.0 kg for adult), respectively (24–26).

The health risk index (HRI) for the population of study area through the consumption of cow’s milk was assessed in accordance with equation 2 (20–22):

(2) $$\mathrm{HRI}=\frac{\text{DIM}}{\text{RfD}}$$,

where DIM and RfD are daily intake of metal and oral reference dose of metal, respectively. The RfD for Cd, Cu, Pb, and Zn were 1.0, 40.0, 3.50, and 330.0 μg/kg/day, respectively. Here, if the HRI < 1, is assumed the exposed populations to be safe (19, 22, 26).

The total HRI (THRI) of heavy metals for the milk was calculated according to equation 3 (27):

(3) THRI=HRI (toxicant 1)+HRI (toxicant 3)+…+HRI (toxicant n)

### Statistical Analyses

The statistical analysis of the obtained results consisted in a first Shapiro-Wilk test for normality, followed by the study of the one-way ANOVA to examine statistical significance of differences in the mean concentration of analyzed metals in milk samples. Moreover, concentration of the analyzed metals in cow’s milk between raw and pasteurized samples was compared by the independent t-test. Finally, to study a correlation between the metals in the different cow’s milk samples, 2-tailed test of Pearson correlation was performed.

## Results

The contents of Cd, Cu, Pb and Zn in the milk samples are presented in Table 1.

**Table 1:** Heavy metal concentrations (mean ± S.D.) of the milk samples (μg/kg, DW)

***Sample***	***Metal Concentration***			
	***Cd***	***Cu***	***Pb***	***Zn***
***Raw Milk***				
1	0.78±0.01^e*^	15.50±0.50^e^	66.90±0.43^e^	364.0±0.00^e^
2	0.78±0.01^e^	15.90±0.72^e^	67.30±0.64^e^	364.0±0.58^e^
3	0.78±0.01^e^	16.60±0.06^f^	68.00±0.00^f^	364.0±0.00^e^
4	0.45±0.02^d^	8.00±0.00^b^	28.00±0.15^d^	278.0±0.00^d^
5	0.42±0.01^c^	8.00±0.06^b^	28.60±0.02^d^	278.0±0.58^d^
6	0.44±0.01^d^	8.00±0.00^b^	29.00±0.00^d^	278.0±0.00^d^
7	0.06±0.01^a^	9.20±0.21^c^	19.00±0.02^b^	252.0±1.00^c^
8	0.06±0.01^a^	9.10±0.61^c^	19.20±0.55^b^	249.0±4.73^b^
9	0.06±0.01^a^	10.00±0.02^d^	19.80±0.26^c^	251.7±0.58^bc^
10	0.18±0.01^b^	5.70±0.00^a^	15.70±0.06^a^	122.0±0.00^a^
11	0.18±0.01^b^	5.70±0.06^a^	15.70±0.17^a^	122.0±0.00^a^
12	0.18±0.00^b^	5.70±0.06^a^	15.90±0.06^a^	122.0±0.00^a^
**Mean Concentration±S.D.**	0.36±0.28	9.77±3.91	32.83±20.80	253.70±87.96

***Pasteurized Milk***				
1	0.01±0.00^a^	5.10±0.00^bc^	1.10±0.00^b^	60.10±0.00^d^
2	0.01±0.00^a^	5.10±0.00^bc^	1.10±0.00^b^	60.00±0.05^d^
3	0.01±0.00^a^	5.10±0.00^bc^	1.10±0.03^c^	61.00±0.00^e^
4	0.20±0.00^a^	5.00±0.00^abc^	1.00±0.00^a^	30.20±0.29^b^
5	0.20±0.00^a^	5.00±0.00^abc^	1.00±0.00^a^	30.20±0.29^b^
6	0.14±0.10^a^	5.00±0.06^bc^	1.10±0.00^b^	31.00±0.00^c^
7	21.20±0.29^c^	18.50±0.10^d^	63.0±0.00^e^	25.00±0.00^a^
8	21.30±0.58^c^	18.40±0.26^d^	63.0±0.00^e^	25.00±0.00^a^
9	22.00±0.00^d^	18.90±0.10^d^	63.0±0.00^e^	25.00±0.00^a^
10	0.60±0.00^b^	4.30±0.58^a^	37.0±0.00^d^	244.70±0.57^fg^
11	0.59±0.00^b^	4.70±1.15^ab^	37.0±0.00^d^	244.30±0.57^f^
12	0.59±0.00^b^	5.70±0.10^c^	37.0±0.00^d^	245.00±0.00^g^
**Mean Concentration±S.D.**	5.57±9.33	8.41±5.99	25.54±26.50	90.12±91.52

* The letters (a, b, c, d …) represent the significant difference between the mean concentration of metals in cow’s milk samples that computed by One-way ANOVA and Duncan multiple range test (p = 0.05)

Based on the results, among the raw cow’s milk samples, Cd, Cu, Pb and Zn (μg/kg) were detected in amount ranging from 0.06 to 0.78, 5.70 to 16.60, 15.70 to 68.0, and 122.0 to 364.0, respectively. In addition, among the pasteurized cow’s milk samples, Cd, Cu, Pb and Zn (μg/kg) were detected an amount ranging from 0.01 to 22.0, 4.30 to 18.90, 1.0 to 63.0, and 25.0 to 245.0, respectively.

Comparing the heavy metal contents in milk samples with the maximum permissible limits (MPL) (2.6 μg/kg for Cd, 10 μg/kg for Cu, 20 μg/kg for Pb and 328 μg/kg for Zn, respectively.) established by International Dairy Federation (28), indicated that the mean concentration of Cd in raw cow’s milk samples and Cu and Zn in raw and pasteurized cow’s milk samples, were lower than MPL.

In addition, HRI values in adults and children through consumption of both raw and pasteurized cow’s milk were lower than 1 (safe limits) (Table 2). Furthermore, the total HRI values (THRI) of heavy metals for adults via consumption of raw cow’s milk, which varied from 8.66E-04 to 3.71E-03 and for children were varied from 4.04E-03 to 1.73E-02 and below the safe limit (THRI < 1). Besides, the THRI values for adults through consumption of pasteurized cow’s milk, which varied from 8.28E-05 to 7.02E-03 and for children, were varied from 1.21E-02 to 3.27E-02 and similar to raw milk samples below the safe limit (THRI < 1). Therefore, all consumers including adults and children have no potential health risk through consuming raw and pasteurized cow’s milk from the study area.

**Table 2:** Daily intakes of metals (DIM, μg) and health risk index (HRI) for individual heavy metal caused by the raw and pasteurized cow's milk

	*Cd*	*Cu*	*Pb*	*Zn*
***Raw Milk***				

Adults				
DIM	6.12E-05	1.66E-03	5.58E-03	3.10E-01
STD	4.76E-05	6.65E-04	3.54E-03	1.50E-02
Min	1.02E-05	9.69E-04	2.67E-03	2.10E-02
Max	1.33E-04	2.82E-03	1.16E-02	6.20E-02
HRI	6.12E-05	4.15E-05	1.59E-03	1.03E-03
STD	4.76E-05	1.66E-05	1.01E-03	4.98E-05
Min	1.02E-05	2.42E-05	7.62E-04	6.91E-05
Max	1.33E-04	7.05E-05	3.30E-03	2.10E-04
Children				
DIM	2.86E-04	7.75E-03	2.60E-02	2.01E-01
STD	2.22E-04	3.10E-03	1.65E-02	6.98E-02
Min	4.76E-05	4.52E-03	1.24E-02	9.68E-02
Max	6.20E-04	1.32E-02	5.39E-02	2.89E-01
HRI	2.86E-04	1.94E-04	7.44E-03	6.71E-04
STD	2.22E-04	7.75E-05	4.71E-03	2.33E-04
Min	4.76E-05	1.13E-04	3.56E-03	3.22E-04
Max	6.20E-04	3.29E-04	1.54E-02	9.62E-04

***Pasteurized Milk***				
Adults				
DIM	9.47E-04	1.43E-03	4.34E-03	1.53E-02
STD	1.59E-03	1.02E-03	4.51E-03	1.55E-02
Min	1.70E-06	7.31E-04	1.70E-04	4.25E-04
Max	3.74E-03	3.21E-03	1.07E-04	4.16E-02
HRI	9.46E-04	3.57E-05	1.24E-03	5.10E-05
STD	1.59E-03	2.54E-05	1.29E-03	5.19E-05
Min	1.70E-06	1.83E-05	4.86E-05	1.42E-05
Max	3.74E-03	8.03E-05	3.06E-03	1.39E-04
Children				
DIM	4.42E-03	6.67E-03	2.03E-02	7.15E-02
STD	7.40E-03	4.75E-03	2.10E-02	7.26E-02
Min	7.93E-06	4.72E-01	7.93E-04	1.98E-02
Max	1.74E-02	1.50E-02	5.00E-02	1.94E-01
HRI	4.42E-03	1.67E-04	5.79E-03	2.38E-04
STD	7.40E-03	1.19E-04	6.00E-03	2.42E-04
Min	7.93E-06	1.18E-02	2.27E-04	6.61E-05
Max	1.74E-02	3.75E-04	1.43E-02	6.47E-04

Based on the results of the Pearson’s correlations coefficient in the raw and pasteurized milk samples, there are significantly correlated were found between concentrations of some metals.

The results of independent t-test showed that there are significantly different (*P* < 0.05) were found in the contents of Cd and Zn between the raw and pasteurized cow’s milk samples. While, for the levels of Cu and Pb no significantly differences (*P* > 0.05) between the raw and pasteurized cow’s milk samples were found.

## Discussion

In this study, the 36 raw and 36 pasteurized milk samples were analyzed for determination of Cd, Cu, Pb and Zn content after the microwave acid extraction. Cadmium is very toxic metal with a natural occurrence or human activities origin in the environment (29). Acute toxicities of Cd cause defection of cardiovascular and skeleton systems (8). The mean concentrations of Cd in raw and pasteurized cow’s milk samples (μg/kg) were 0.36±0.28 and 5.57±9.33, respectively and that was higher than the recommended standards were found in pasteurized cow’s milk samples. Cadmium accumulation in plants as the animal feed grown near industrial areas, zones of storage of mining waste depots and regions with high traffic volume can translocation the Cd in the animals’ tissues, and consequently in the milk (1, 30, 31). Moreover, except for raw materials, the equipment used during the production process and packaging materials are the possible sources of contamination (5). The obtained results are similar to the findings that concluded the concentrations of Cd in raw milk samples collected from Serbia (3.50 μg/kg) were much lower than Serbian regulations maximal permitted contents (10 μg/kg) (5). Comparison of our findings with other studies is shown in Table 3.

**Table 3:** The metal contents (μg/kg) in raw and pasteurized milk samples compared to other studies

***Region***	***Elements***				***References***
	**Cd**	**Cu**	**Pb**	**Zn**	
Iran (Raw milk)	0.36	9.77	32.83	253.70	Present study
Iran (Pasteurized Milk)	5.57	8.41	25.54	90.12	Present study
Egypt	246	1448	3141	7203	(28)
Poland (Simmental cow)	3.50	37.70	36.60	3027	(42)
Poland (Holstein-Friesian cow)	4.00	45.30	41.20	3277	(42)
Spain	<2	-	3.91	-	(1)
South Africa	<0.006	-	5.44	-	(32)
Lithuania (winter season)	0.37	-	0.47	-	(33)
Lithuania (Summer season)	0.18	-	0.54	-	(33)
Croatian	5.31	-	34.00	-	(3)
Iran (Hamadan Province)	3.21	-	4.84	-	(34)
Iran (Raw Milk)	2.87	-	60.72	-	(35)
Iran (Pasteurized Milk)	1.03	-	13.57	-	(35)
Iran (Raw Milk)	104.00	142.00	2720.00	3072.00	(36)
China	305.00	-	-	-	(37)
Pakistan (Area 1)	89.00	-	21781	-	(38)
Pakistan (Area 2)	62.00	-	15958	-	(38)
Denmark	0.66	-	-	-	(39)

Copper known as essential micronutrients for growing plants should be ensured through organic and artificial fertilizers or which is important to healthy hormone secretion, nerve conduction, and the growth of bones and connective tissue. As the literature shows, Cu plays a critical role in some biochemical processes (9, 10). Based on the results, the concentrations of Cu in raw and pasteurized cow’s milk samples (μg/kg) with an average of 9.77±3.91 μg/kg and 8.41±5.99 μg/kg were lower than the MPL. This reported of Cu level was much lower than those observed in raw milk samples collected from Serbia (117.70 μg/kg) (5). Although, the mean concentrations of Cu were below the MPL, animal feed, drinking water with the high amount of Cu and Cu alloys used in equipment is a possible source of contamination of Cu in milk. (28). Comparison of our findings with other studies is shown in Table 3.

Lead as a highly toxic metal has no known functions in biological systems and recognized as a major environmental health risk throughout the world (40). The concentrations of Pb in raw and pasteurized cow’s milk samples (μg/kg) with an average of 32.83±20.80 and 25.54±26.50, respectively were higher than the recommended standards. Similarly, like for Cd, the highest Pb levels in milk samples were reported in animals reared in the vicinity of Pb–Zn smelter, mining waste depots, thermal power and roads usually exposed to more automotive Pb emissions (1). In this regard, Suturovic et al. (2014) reported that the Pb contents in raw milk samples were 75.42 μg/kg. Comparison of our findings with other studies is shown in Table 3.

Zinc is known as vital element for human growth (14). The concentrations of Zn in raw and pasteurized cow’s milk samples (μg/kg) with an average of 253.70±87.96 and 90.12±91.52, respectively were lower than the MPL. These values were lower than findings that reported the variation in Zn content in milk samples collected from Egypt ranged from 888 μg/kg to 18316 μg/kg (41). Comparison of our findings with other studies is shown in Table 3.

The average HRI values through consumption of raw cow’s milk were 6.81E-04 and 2.15E-03 for adults and children, respectively (Table 2). While, the average HRI values through consumption of pasteurized cow’s milk were 5.68E-04 and 2.65E-03 for adults and children, respectively. Therefore, mean of HRI values of analyzed metals for adults and children through consumption of raw and pasteurized cow’s milk are lower than 1. In this regard, the HRI of all analyzed metals were minimal.

The Pearson’s correlations coefficient were performed between metal concentrations in raw and pasteurized cow’s samples to understand the relationships between them. In the raw milk samples the residual content of Cd was significantly positively correlated with the contents of Cu (r=0.779), Pb (r=0.939) and Zn (r=0.760), the residual content of Cu was significantly positively correlated with the concentrations of Pb (r=0.941) and Zn (r=0.884) and the residual content of Pb was significantly positively correlated with the concentration of Zn (r=0.842). Moreover, in the pasteurized milk samples the residual content of Cd was significantly positively correlated with the concentrations of Cu (r=0.997) and Pb (r=0.840) and negatively correlated with the concentration of Zn (r=−0.397), and the residual content of Cu was significantly positively correlated with the concentration of Pb (r=0.819) and the negative correlated with the concentration of Zn (r=−0.425), proved in our work. In this regard, strong positive correlations were reported between Pb and Cd concentrations (r=0.85 vs. r=0.87) in the milk of Simmental and Holstein-Friesian breeds (42). The residual content of Cu in milk was significantly positively correlated with the concentrations of Zn (r=0.629) (43). However, significant negative correlations were found between Cu and Zn concentrations (r=−0.377) (44).

## Conclusion

This study was carried out to analyze the Cd, Cu, Pb, and Zn contents in raw and pasteurized cow’s milk from city of Hamadan, Iran. Based on the results, although in the pasteurized cow’s milk samples, the contents of Cu, Pb, and Zn were lower compared to the raw cow’s milk samples, but considering the serious contamination of three samples of pasteurized milk by Cd, four samples of raw milk and three samples of pasteurized milk by Cu, six samples of both raw and pasteurized milk by Pb and three samples of raw milk by Zn (above the MPL) a control of heavy metals content during the whole production process of milk must be applied. In addition, to maintain human health, it is recommended that special attention be given to the adverse effect of heavy metals through the consumption of other foodstuffs.

## Ethical considerations

Ethical issues (Including plagiarism, informed consent, misconduct, data fabrication and/or falsification, double publication and/or submission, redundancy, etc.) have been completely observed by the author.
